# Dietary phytosterols and phytostanols decrease cholesterol levels but increase blood pressure in WKY *inbred *rats in the absence of salt-loading

**DOI:** 10.1186/1743-7075-7-11

**Published:** 2010-02-12

**Authors:** Qixuan Chen, Heidi Gruber, Eleonora Swist, Kara Coville, Catherine Pakenham, Walisundera MN Ratnayake, Kylie A Scoggan

**Affiliations:** 1 Nutrition Research Division, Food Directorate, Health Products and Food Branch, Health Canada, Banting Research Centre, Ottawa, Ontario K1A 0K9, Canada; 2 Department of Biochemistry, Microbiology and Immunology, University of Ottawa, Ottawa, Ontario K1H 8M5, Canada

## Abstract

**Background:**

There are safety concerns regarding widespread consumption of phytosterol and phytostanol supplemented food products. The aim of this study was to determine, in the absence of excess dietary salt, the individual effects of excess accumulation of dietary phytosterols and phytostanols on blood pressure in Wistar Kyoto (WKY) *inbred *rats that have a mutation in the *Abcg5 *gene and thus over absorb phytosterols and phytostanols.

**Methods:**

Thirty 35-day old male WKY *inbred *rats (10/group) were fed a control diet or a diet containing phytosterols or phytostanols (2.0 g/kg diet) for 5 weeks. The sterol composition of the diets, plasma and tissues were analysed by gas chromatography. Blood pressure was measured by the tail cuff method. mRNA levels of several renal blood pressure regulatory genes were measured by real-time quantitative PCR.

**Results:**

Compared to the control diet, the phytosterol diet resulted in 3- to 4-fold increases in the levels of phytosterols in plasma, red blood cells, liver, aorta and kidney of WKY *inbred *rats (*P *< 0.05). The phytostanol diet dramatically increased (> 9-fold) the levels of phytostanols in plasma, red blood cells, liver, aorta and kidney of these rats (*P *< 0.05). The phytosterol diet decreased cholesterol levels by 40%, 31%, and 19% in liver, aorta and kidney, respectively (*P *< 0.05). The phytostanol diet decreased cholesterol levels by 15%, 16%, 20% and 14% in plasma, liver, aorta and kidney, respectively (*P *< 0.05). The phytostanol diet also decreased phytosterol levels by 29% to 54% in plasma and tissues (*P *< 0.05). Both the phytosterol and phytostanol diets produced significant decreases in the ratios of cholesterol to phytosterols and phytostanols in plasma, red blood cells, liver, aorta and kidney. Rats that consumed the phytosterol or phytostanol diets displayed significant increases in systolic and diastolic blood pressure compared to rats that consumed the control diet (*P *< 0.05). The phytosterol diet increased renal *angiotensinogen *mRNA levels of these rats.

**Conclusion:**

These data suggest that excessive accumulation of dietary phytosterols and phytostanols in plasma and tissues may contribute to the increased blood pressure in WKY *inbred *rats in the absence of excess dietary salt. Therefore, even though phytosterols and phytostanols lower cholesterol levels, prospective clinical studies testing the net beneficial effects of dietary phytosterols and phytostanols on cardiovascular events for subgroups of individuals that have an increased incorporation of these substances are needed.

## Background

Elevated blood levels of total cholesterol and low-density lipoprotein cholesterol (LDL-C) are established risk factors for the development of cardiovascular disease in humans [1]. Phytosterols and phytostanols are lipid compounds structurally similar to cholesterol. Intake of phytosterols and/or phytostanols at the level of 1.5-3.0 g/day has been documented to reduce blood LDL-C by 10% [2,3], which would decrease coronary heart disease risk by 27%, if the LDL-C reduction was sustained for 5 years [4]. The efficacy and safety of phytosterol- and phytostanol-enriched food products has been reviewed by several regulatory agencies, and the sale of phytosterol- and phytostanol-enriched food products have been allowed as a means to reduce blood cholesterol levels by many European countries [5], the United States [6], and Australia and New Zealand [7]. In Canada, health claims for phytosterol and phytostanol food supplementation are currently under review [8].

No unexpected side effects have been reported since phytosterol- and phytostanol-enriched food products have been introduced into the food supply [9]. However, studies indicating a pro-atherogenic effect of phytosterols and phytostanols have been published, as outlined below. First, phytosterolemic patients over-absorb and accumulate phytosterols and phytostanols, and develop premature coronary artery disease [10,11]. Second, the use of phytosterol or phytostanol-enriched food products was found to elevate phytosterol and phytostanol levels in serum in free-living populations [12,13] and in healthy subjects [14], and epidemiological studies have demonstrated that elevated serum levels of phytosterols and phytostanols are associated with increased atherosclerotic cardiovascular disease [15-18]. Third, phytosterols and phytostanols have been detected in atherosclerotic lesions from a phytosterolemic patient who died suddenly of a myocardial infarction [10], and also from patients with aortic stenosis [19]. Fourth, phytosterol and phytostanol supplementation increased plasma phytosterol and phytostanol levels, impaired endothelial function and increased cerebral lesion size in wild-type mice [19]. Fifth, elevated systolic blood pressure was observed in salt-loaded spontaneously hypertensive-stroke prone (SHRSP), spontaneously hypertensive (SHR) and Wistar Kyoto (WKY) rats fed a high phytosterol diet [20-24], as well as in a phytosterolemic patient [10].

Although phytosterolemia (also known as sitosterolemia) is a rare autosomal recessive disorder, the prevalence of this disorder is likely underestimated due to misdiagnosis as hyperlipidemia since the standard colorimetric and enzymatic methods for the detection of cholesterol do not distinguish cholesterol from phytosterols and phytostanols. In addition, although homozygosity for a mutation that causes phytosterolemia is rare, heterozygosity is much more common (1/1100 individuals) [3]. These individuals usually have 2-fold higher levels of serum phytosterols compared to healthy people [25,26]. In a study of 595 hypercholesterolemic subjects in the United States, 3.5% of the subjects had elevated levels of serum phytosterols with accelerated atherosclerosis [15]. These results suggest that a substantial proportion of the population that would be recommended to consume phytosterol and phytostanol supplemented foods could be made worse by these products. Therefore, although most healthy individuals do not absorb high levels of phytosterols and phytostanols, it is important to try to understand the mechanisms of action of phytosterols and phytostanols for subgroups of individuals that have an increased incorporation of these substances. Also, since phytosterol and phytostanol supplemented foods are being marketed for individuals with high serum cholesterol, a risk factor for heart disease, it is important to assess the mechanisms of action of these products for individuals predisposed to coronary heart disease, stroke and atherosclerosis.

Our previous data showed that dietary phytosterols and phytostanols increased the incorporation of phytosterols and phytostanols in plasma and tissues in diabetic [27] and salt-loaded SHRSP rats [28-30] as well as elevated diastolic blood pressure, accelerated the onset of stroke and reduced the life span in salt-loaded SHRSP rats [28-31]. However, salt-loading is known to accelerate the development of hypertension and hypertensive-related conditions [32]. The presence of extra salt may contribute to or mask effects of dietary phytosterols and phytostanols. In the present study our objective was to isolate and identify the individual effects of increased intake of phytosterols and phytostanols on sterol accumulation, blood pressure and the expression of renal blood pressure regulatory genes from any potential confounding effects of salt-loading. WKY *inbred *rats were used because this rat strain is usually used as a 'normotensive' model compared to its 'descendant' SHRSP rat strain [33]. Also, we have determined that, similar to phytosterolemic patients, WKY *inbred *rats have a mutation in the *Abcg5 *gene that results in increased tissue incorporation of phytosterols and phytostanols [34]. Therefore, we can use this model to investigate the health effects and mechanisms of action that result from increased absorption of dietary phytosterols and phytostanols.

## Materials and methods

### Phytosterols and phytostanols preparation

Phytosterols were prepared from canola oil deodorizer distillate as described previously by us [29]. Deodorizer distillate is a by-product of the deodorization of vegetable oils. It is a complex mixture of free fatty acids, phytosterols and their esters, tocopherol esters, and mono-, di- and triacylglycerols. In a typical preparation, 1 kg of canola oil deodorizer distillate (supplied by CanAmera Foods, Altona, Manitoba, Canada) containing 19.9% sterols was saponified, acidified and methylated. The non-saponifiable fraction containing free phytosterols was recovered and recrystalized using heptane. This produced 124 g of 100% pure phytosterols. The purity of the phytosterol product was determined by gas liquid chromatography (GC) and thin-layer chromatography analysis (TLC) [29]. The phytosterol product contained 22.0% brassicasterol, 31.9% campesterol, 43.2% beta-sitosterol and 2.9% other phytosterols.

A portion of the recrystalized phytosterol product was converted to phytostanols via catalytic hydrogenation as described previously by us [31] at the pilot plant facility of the POS Plant Corporation (Saskatoon, SK, Canada). The final phytostanol product was 100% pure as determined by GC and TLC, and contained 54.7% campestanol and 44.8% sitostanol.

### Diet preparation

Three diets were prepared according to our previous publication [31] based on the AIN-93G recommendations for rodent diets [35]. The final total phytosterol and phytostanol content of the diets was 0.2, 2.1 and 1.9 g/kg in the control diet, phytosterol diet and phytostanol diet, respectively. These levels are the same as the levels we used in our previous study [31]. These dietary levels of phytosterols and phytostanols were used because our previous studies demonstrated that 2.0 g/kg diet of phytosterols and phytostanols significantly accelerated the onset of stroke and reduced the life span of SHRSP rats [28-30]. These dietary levels of phytosterols and phytostanols are similar to the amounts suggested for cholesterol lowering in humans on a dietary fat basis [3]. For example, consumption of 2.0 g per day of dietary phytosterols or phytostanols in an individual that consumes 100 g of fat per day (average fat intake) represents 2.0% of that person's fat intake. Our rats were also fed 2.0 g of phytosterols or phytostanols in a diet that contained 100 g of fat (soybean oil) per kg of diet which represents 2.0% of the rat's dietary fat intake.

### Animals

WKY *inbred *rats were obtained from Charles River Laboratories (Kingston, NY, USA). 35-day old male WKY *inbred *rats were randomly assigned to one of three diet groups (n = 10/group, Table 1). They had free access to drinking water. After 5 weeks on the test diets, 12 h food-deprived rats were euthanised via exsanguination from the abdominal aorta while under 3% isoflurane anaesthesia. Blood was collected in heparinised polystyrene tubes. Plasma and red blood cells were isolated as described previously [28]. Liver, aorta and kidneys were removed, frozen immediately in liquid nitrogen and stored at -80°C. The animal protocol was approved by the Institutional Animal Care Committee at Health Canada.

**Table 1:** Composition of test diets ^1^

	Control diet	Phytosterol diet	Phytostanol diet
**Ingredient**	*g/kg diet*		
Vitamin free casein	222.0	222.0	222.0
Soybean oil	100.0	100.0	100.0
Phytosterols ^2^	---	2.0	---
Phytostanols ^3^	---	---	2.0
Cornstarch	477.5	475.5	475.5
Granulated sugar	100.0	100.0	100.0
Cellulose (alphacel)	50.0	50.0	50.0
Miscellaneous ^4^	50.5	50.5	50.5
			
**Sterol composition**			
Cholesterol	0.0	0.0	0.0
Total phytosterols ^5^	0.2	2.1	0.2
Total phytostanols ^6^	0.0	0.0	1.7
Total phytosterols and phytostanols	0.2	2.1	1.9

^1^Based on the AIN-93G diet (American Institute of Nutrition, 1993). All ingredients, except phytosterols and phytostanols, were purchased from Harlan Teklad (Madison, WI).

^2^Phytosterols isolated from canola oil deodorizer distillate (CanAmera Foods, Altona, MB, Canada).

^3^Phytostanols were hydrogenated from phytosterols (POS Pilot Plant Corporation, Saskatoon, SK, Canada).

^4^93G-MX Mineral mix (35.0 g/kg), 93-VX Vitamin mix (10.0 g/kg), L-cystine (3.0 g/kg), choline bitartrate (2.5 g/kg) and tert-butylhydroquinone (0.014 g/kg).

^5^Total phytosterols include campesterol, beta-sitosterol, stigmasterol, brassicasterol, and delta 5,24- stigmastadienol.

^6^Total phytostanols include campestanol and sitostanol.

### Analyses of sterols

The sterol composition of the diets, plasma, red blood cells, liver, aorta, and kidney were analysed by gas chromatography as described previously [29,30].

### Measurement of blood pressure

Blood pressure was measured at the beginning, middle and end (week 1, 3 and 5) of the feeding phase of the study by the tail cuff method using a II TC Model 31 Blood Pressure Apparatus (II TC INC/Life Science Instruments, Woodland Hills, CA, USA) as described previously [31]. Briefly, the blood pressure apparatus was stored in a separate, warmer room (26-27°C). The pressure channels were calibrated every day and the chamber was kept at 29-30°C. Rats were trained on the apparatus every day for 1 week prior to the experiment. Rats were transferred to the blood pressure room 30 min before handling to allow them to gradually get used to the heat. Each rat was placed in the chamber for 5 min prior to starting the measurements, allowing rats to acclimatize. The same investigator monitored the computer screen and the animal in order to detect artifacts such as animal distress or movement. The systolic blood pressure and the mean blood pressure were measured by the same investigator every time. The diastolic blood pressure was calculated by the equation: diastolic = (3 × mean - systolic)/2 as per the manufacturer's instructions. The blood pressure of each rat at each measuring point was obtained as an average of three readings. The coefficient of variation (CV) is from 1.47% to 6.24%.

### RNA isolation and real-time quantitative RT-PCR

Procedures described previously were followed [31]. Briefly, total RNA was extracted from each kidney sample and purified. Total RNA (1 μg) was reverse-transcribed to synthesize cDNA with Retroscript Kit (Ambion, Austin, TX, USA). Real-time quantitative PCR was performed on a Mx4000 Multiplex Quantitative PCR System using the Brilliant SYBR Green QPCR Core Reagent Kit (Stratagene, La Jolla, CA, USA) or Taqman Gene Expression Assay (Applied Biosystems, Foster city, CA, USA) as described previously [31].

### Statistical analysis

Data are presented as the means ± standard deviation (SD). Statistical analyses were performed using Statistica 8.0 software (StatSoft, Tulsa, OK, USA). One-way analysis of variance (ANOVA) followed by post-hoc Duncan's multiple range test was used to determine the diet effects and compare differences among group means. All data were evaluated for equality of variance prior to statistical analysis. Variables with skewed distribution were logarithm-transformed. A non-parametric Kruskal-Wallis test followed by Wilcoxon rank sum tests was employed if the data failed to satisfy equality of variance. Differences were considered significant when *P *≤ 0.05.

## Results

### Body weight, food intake, water consumption and histopathologic findings

Body weight of the animals increased steadily during the 5 week feeding phase of the study. There were no effects of diet on body weight gain, food efficiency and total water consumption (Table 2). No significant lesions were found in liver and kidney in paraffin-embedded hematoxylin and eosin stained slides. A cross-section of thoracic and abdominal aorta was examined and no significant lesions were seen (data not shown).

**Table 2:** Growth, food and water intake of the rats in the absence of salt-loading

	Control diet (n = 10)	Phytosterol diet (n = 10)	Phytostanol diet (n = 10)	*P *value (one-way ANOVA)
Initial body wt (g)	131.5 ± 15.2	133.9 ± 13.5	129.2 ± 12.7	0.7513
Body wt at week 5 (g)	265.9 ± 26.3	267.3 ± 16.0	258.5 ± 19.5	0.6084
Weight gain (g/5 weeks)	134.4 ± 13.0	133.5 ± 5.8	129.4 ± 8.8	0.4728
Total food intake (g)	539.6 ± 55.6	541.2 ± 46.6	519.0 ± 33.7	0.4959
Food efficiency (g gain/kg intake)	249.3 ± 8.6	247.9 ± 18.1	249.4 ± 10.3	0.9590
Total water consumption (g)	1010.9 ± 197.8	963.7 ± 82.5	1045.1 ± 147.9	0.4872*

Values are presented as means ± SD. * Nonparametric (Kruskal-Wallis) test

### Sterol composition in plasma, red blood cells, liver, aorta, and kidney

In comparison to the control diet, the phytosterol diet increased phytosterol incorporation in plasma (4.1-fold, *P *< 0.05), red blood cells (3-fold, *P *< 0.05), liver (2.9-fold, *P *< 0.05), aorta (3.1-fold, *P *< 0.05) and kidney (4.1-fold, *P *< 0.05) in WKY *inbred *rats (Table 3). The phytostanol diet dramatically increased phytostanol incorporation in plasma (145-fold, *P *< 0.05), red blood cells (9.2-fold, *P *< 0.05), liver (31.6-fold, *P *< 0.05), aorta (95-fold, *P *< 0.05) and kidney (141-fold, *P *< 0.05) in these rats. The phytostanol diet decreased phytosterol incorporation in plasma (54%, *P *< 0.05), red blood cells (35%, *P *< 0.05), liver (29%, *P *< 0.05), aorta (38%, *P *< 0.05) and kidney (46%, *P *< 0.05).

**Table 3:** Sterol levels in plasma (mg/100 ml) and tissues (mg/100 g) of rats in the absence of salt-loading

Tissue	Sterol	Control diet (n = 10)	Phytosterol diet (n = 10)	Phytostanol diet (n = 10)	*P *value (one-way ANOVA)
Plasma					
	Total phytosterols	8.2 ± 1.3^b^	33.6 ± 4.9^c^	3.8 ± 0.7^a^	0.0001
	Total phytostanols	0.1 ± 0.1^a^	0.1 ± 0.1^a^	14.5 ± 2.2^b^	0.0001*
	Cholesterol	80.9 ± 5.8^b^	75.5 ± 10.3^ab^	68.8 ± 6.9^a^	0.0076
	Total sterols	89.1 ± 6.6^a^	109.1 ± 14.1^b^	87.1 ± 9.0^a^	0.0001
	Cholesterol/phytosterols & phytostanols	10.0 ± 1.3^c^	2.3 ± 0.3^a^	3.8 ± 0.4^b^	0.0001
Red blood cells					
	Total phytosterols	10.0 ± 2.6^b^	29.7 ± 7.4^c^	6.5 ± 0.7^a^	0.0001
	Total phytostanols	1.0 ± 0.4^a^	0.8 ± 0.4^a^	9.2 ± 0.9^b^	0.0001
	Cholesterol	101.9 ± 26.1	81.9 ± 20.8	85.2 ± 10.8	0.0789
	Total sterols	112.9 ± 28.5	112.5 ± 28.3	100.9 ± 11.3	0.4611
	Cholesterol/phytosterols & phytostanols	9.4 ± 1.5^c^	2.7 ± 0.2^a^	5.5 ± 0.8^b^	0.0001
Liver					
	Total phytosterols	19.0 ± 5.3^b^	54.8 ± 8.1^c^	13.5 ± 1.5^a^	0.0001
	Total phytostanols	0.7 ± 0.2^a^	0.6 ± 0.3^a^	22.1 ± 2.7^b^	0.0001
	Cholesterol	223.5 ± 46.1^c^	134.3 ± 18.1^a^	187.9 ± 23.7^b^	0.0001
	Total sterols	243.3 ± 51.1^b^	189.8 ± 24.6^a^	223.6 ± 27.0^b^	0.0141
	Cholesterol/phytosterols & phytostanols	11.5 ± 1.1^c^	2.4 ± 0.2^a^	5.3 ± 0.4^b^	0.0001
Aorta					
	Total phytosterols	8.7 ± 2.9^b^	26.6 ± 5.7^c^	5.4 ± 0.9^a^	0.0001
	Total phytostanols	0.1 ± 0.1^a^	0.3 ± 0.7^a^	9.5 ± 1.8^b^	0.0001*
	Cholesterol	125.5 ± 32.8^c^	86.5 ± 20.4^a^	100.7 ± 14.0^ab^	0.0038
	Total sterols	134.1 ± 35.4^b^	113.4 ± 25.3^a^	115.5 ± 13.2^a^	0.1705
	Cholesterol/phytosterols & phytostanols	14.7 ± 2.1^c^	3.2 ± 0.5^a^	7.0 ± 2.0^b^	0.0001
Kidney					
	Total phytosterols	9.8 ± 1.8^b^	40.2 ± 3.8^c^	5.3 ± 0.8^a^	0.0001
	Total phytostanols	0.1 ± 0.1^a^	0.1 ± 0.1^a^	14.1 ± 1.2^b^	0.0001*
	Cholesterol	320.6 ± 10.4^b^	258.6 ± 28.6^a^	276.2 ± 46.6^a^	0.0006
	Total sterols	330.4 ± 10.9^b^	298.8 ± 27.8^a^	295.7 ± 47.3^a^	0.0438
	Cholesterol/phytosterols & phytostanols	33.6 ± 5.2^c^	6.5 ± 1.1^a^	14.2 ± 2.4^b^	0.0001

Values are presented as means ± SD. * Nonparametric (Kruskal-Wallis) test

^a,b,c ^means in a row not sharing a superscript letter are significantly different (*P *< 0.05, Duncan's test or Wilcoxon rank sum tests).

Total phytosterols include campesterol, beta-sitosterol, stigmasterol, brassicasterol, and delta 5,24-stigmastadienol.

Total phytostanols include campestanol and sitostanol.

Total sterols include cholesterol + total phytosterols + total phytostanols.

In comparison to the control diet, the phytosterol diet decreased cholesterol levels in liver (40%, *P *< 0.05), aorta (31%, *P *< 0.05) and kidney (19%, *P *< 0.05) of these rats. The phytostanol diet also decreased cholesterol levels in plasma (15%, *P *< 0.05), liver (16%, *P *< 0.05), aorta (20%, *P *< 0.05) and kidney (14%, *P *< 0.05). The effects of phytosterols and phytostanols on cholesterol lowering in red blood cells did not reach statistical significance (overall *P *= 0.0789, Table 3).

Compared to the control diet, the phytosterol diet decreased total sterol levels in liver (*P *< 0.05), aorta (*P *< 0.05) and kidney (*P *< 0.05), but increased total sterols in plasma (*P *< 0.05) of these rats. The phytostanol diet also decreased total sterol levels in aorta (*P *< 0.05) and kidney (*P *< 0.05). However, the phytosterol diet resulted in the lowest ratio of cholesterol to total phytosterols and total phytostanols in plasma, red blood cells, liver, aorta and kidney (*P *< 0.05, Table 3). The phytostanol diet also decreased this ratio in plasma and all tissues tested (*P *< 0.05).

### Blood pressure

There were no differences in the initial systolic and diastolic blood pressure among groups (*P *= 0.5857 and *P *= 0.5373, respectively, Table 4). After consuming the diets for 5 weeks, the WKY *inbred *rats fed the phytosterol and phytostanol diets had significantly higher systolic (*P *= 0.0067) and diastolic (*P *= 0.0001) blood pressure as well as greater increases in systolic (*P *= 0.0333) and diastolic (*P *= 0.0021) blood pressure, when compared to the rats fed the control diet. The phytostanol diet caused a greater increase in diastolic blood pressure (*P *< 0.05) compared to the phytosterol diet.

**Table 4:** Systolic and diastolic blood pressure (BP) of the rats in the absence of salt-loading

	Control diet (n = 10)	Phytosterol diet (n = 10)	Phytostanol diet (n = 10)	*P *value (one-way ANOVA)
Initial systolic BP (mmHg)	113.7 ± 7.3	110.9 ± 10.7	115.3 ± 8.8	0.5857
Systolic BP at week 5 (mmHg)	127.0 ± 6.2^a#^	136.0 ± 8.2^b#^	138.6 ± 7.4^b#^	0.0067
Change in systolic BP (mmHg/5 weeks)	13.3 ± 8.5^a^	25.1 ± 12.0^b^	23.4 ± 9.1^b^	0.0333
				
Initial diastolic BP (mmHg)	78.0 ± 3.9	81.0 ± 7.8	79.9 ± 6.1	0.5373
Diastolic BP at week 5 (mmHg)	82.6 ± 4.0^a#^	89.7 ± 5.8^b#^	95.2 ± 5.8^c#^	0.0001
Change in diastolic BP (mmHg/5 weeks)	4.6 ± 3.6^a^	8.7 ± 5.8^b^	15.2 ± 7.1^c^	0.0021

Values are presented as means ± SD.

^a,b,c ^means in a row not sharing a superscript letter are significantly different (*P *< 0.05, Duncan's test).

^# ^means significantly different from initial measurement of systolic or diastolic blood pressure, respectively (week 5 vs initial, *P *< 0.05, t-test).

### Renal mRNA expression of genes involved in blood pressure regulation

In comparison to the control diet, the phytosterol diet significantly increased mRNA levels of renal *angiotensinogen *in WKY *inbred *rats (*P *= 0.0339, Table 5), but did not change renal mRNA levels of *renin*, *angiotensin I converting enzyme 1, 2 (Ace1, Ace2)*, *angiotensin II receptor type 1a (Agtr1a*), *nitric oxide synthase 1, 3 (Nos1, Nos3*), *cyclooxygenase 2*, *Spondin 1 *and *THUMP domain containing 1 *(*P *> 0.05). There were no effects of phytostanols on mRNA expression of all genes measured (*P *> 0.05).

**Table 5:** Kidney gene expression of the rats in the absence of salt-loading

	Control diet (n = 10)	Phytosterol diet (n = 10)	Phytostanol diet (n = 10)	*P *value (one-way ANOVA)
*Angiotensinogen*	1.00 ± 0.12^a^	1.20 ± 0.28^b^	0.95 ± 0.17^a^	0.0339
*Renin*	1.00 ± 0.17	1.05 ± 0.17	1.17 ± 0.37	0.3597
*Angiotensin I converting enzyme (Ace) 1*	1.00 ± 0.13	1.05 ± 0.08	1.11 ± 0.34	0.5948
*Ace 2*	1.00 ± 0.15	1.03 ± 0.22	0.99 ± 0.21	0.8884
*Angiotensin II receptor (Agtr) 1a*	1.00 ± 0.08	0.94 ± 0.11	0.93 ± 0.10	0.3131
*Nitric oxide synthase (Nos) 1*	1.00 ± 0.36	0.84 ± 0.20	1.04 ± 0.29	0.3170
*Nos 3*	1.00 ± 0.11	1.00 ± 0.21	1.07 ± 0.21	0.6882
*Cyclooxygenase 2*	1.00 ± 0.26	1.09 ± 0.31	1.09 ± 0.34	0.7696
*Spondin 1*	1.00 ± 0.07	1.10 ± 0.17	1.01 ± 0.10	0.1988
*THUMP domain 1*	1.00 ± 0.16	0.93 ± 0.20	1.10 ± 0.23	0.2103

Values are presented as means ± SD.

^a,b ^means in a row not sharing a superscript letter are significantly different (*P *< 0.05, Duncan's test).

## Discussion

High blood pressure is a serious condition that can lead to coronary heart disease, stroke, kidney failure, and other health problems [36]. Effects of phytosterols and phytostanols on blood pressure remain controversial. In human studies, consumption of a dietary portfolio (plant sterols, soybean protein, viscous fibres and almonds) for 1 year significantly reduced blood pressure in 66 hyperlipidemic subjects [37]. Intake of a product containing sitostanol and campestanol for 4 weeks slightly decreased systolic blood pressure in 100 healthy men [38]. However, elevated systolic blood pressure was observed in a phytosterolemic patient [10]. In animal studies, in the presence of 1% salt-loading, our previous study demonstrated that, phytosterol or phytostanol supplemented diets (2.0 g/kg diet) induced a significant increase in diastolic blood pressure in SHRSP rats [31]. Similarly, Naito *et al*. [22-24] showed that a diet containing 10% rapeseed oil (0.71 g total phytosterols/kg diet) induced higher systolic blood pressure than a diet containing soybean oil (0.29 g total phytosterols/kg diet) in SHRSP, SHR and WKY rats and thus thought that the higher content of phytosterols in rapeseed oil was related to the increased blood pressure. A diet containing soybean oil fortified with phytosterols (4.5 g/kg diet) for 24 days was also reported to elevate systolic blood pressure in SHRSP rats, but there were no significant differences on systolic blood pressure among diets containing 10% rapeseed oil, 10% soybean oil, and 10% soybean oil supplemented with 0.6 g total phytosterols/kg diet [20]. A study by Chen *et al*. [39] demonstrated that consumption of a sitosterol diet (0.3 g/kg diet) for 6 weeks did not increase blood pressure in wild-type, heterozygous and homozygous (a mutation in *Abcg5*) female SHR *inbred *rats. In the absence of salt-loading, our present study found that dietary phytosterols or phytostanols (2.0 g/kg diet) significantly increased systolic and diastolic blood pressure in WKY *inbred *rats (Table 4). To our knowledge, this was the first study to investigate, in the absence of salt-loading, the effects of dietary phytosterols and phytostanols on blood pressure in rodent models. Our results were consistent with those reported by Naito *et al*. [21] who demonstrated that, in the absence of salt-loading, a diet containing 10% rapeseed oil for 4 weeks induced a higher systolic blood pressure in SHRSP rats compared to a diet containing 10% soybean oil. Taken together, our previous and present results suggest that increased intake of phytosterols or phytostanols may exacerbate hypertension in both hypertensive and normotensive rat models that over absorb phytosterols and phytostanols in the presence or absence of salt.

In order to elucidate the molecular mechanism(s) by which intake of phytosterols and phytostanols increased blood pressure in this rat model, the expression of several renal genes known to be involved in the renin-angiotensin-aldosterone system, nitric oxide, and cyclooxygenase-derived prostanoid pathways were investigated (Table 5). Spondin 1 and THUMP domain containing 1 were also measured because they were recently identified as candidate hypertension genes [40,41]. Our present study demonstrated that, in the absence of salt-loading, the phytosterol diet slightly increased the mRNA expression of *angiotensinogen *(1.2-fold, *P *< 0.05, Table 5). There were no effects of phytosterols and phytostanols on the mRNA expression of *renin*, *angiotensin I converting enzyme (Ace1) 1*, *Ace2*, *angiotensin II receptor 1a (Agtr1a)*, *nitric oxide synthases (Nos) 1*, *Nos3*, *Cyclooxygenase 2*, *Spondin 1 *and *Thump domain 1 *(*P *> 0.05). These results suggest that *Nos*, *Cyclooxygenase 2*, *Spondin 1 *and *Thump domain 1 *pathways do not play a critical role in the blood pressure regulation caused by the intake of phytosterols and phytostanols in WKY *inbred *rats. Also, the phytostanol diet increased blood pressure but did not change the mRNA levels of renal *angiotensinogen*. Therefore, the renin-angiotensin-aldosterone system may not play a leading role in the hypertensive responses caused by phytosterol and phytostanol diets in the absence of salt-loading in WKY *inbred *rats.

Currently there are no data indicating that consumption of phytosterol and phytostanol-enriched foods alters cardiovascular events, but epidemiological studies indicated a controversial association of phytosterols and phytostanols with cardiovascular risk. For example, the results from a cohort study in 1242 subjects older than 65 years in the Netherlands showed that plasma levels of phytosterols were significantly lower in patients with coronary heart disease [42]. A prospective nested case-control study consisting of 373 cases and 758 controls in the United Kingdom indicated that there was no adverse relationship between plasma physiological levels of phytosterols and coronary heart disease risk in apparently healthy individuals [43]. The study from 2542 subjects aged 30 to 67 years in the United States showed no significant difference in plasma sitosterol and campesterol levels between the individuals having a positive and a negative family history of CHD [44]. However, studies in 595 subjects with hypercholesterolemia [15], in 48 postmenopausal women [16], in 53 patients undergoing coronary artery bypass graft surgery [17] and in 159 men that suffered a myocardial infarction in the Prospective Cardiovascular Munster study [18] suggest an association of elevated levels of phytosterols and phytostanols with an increased cardiovascular disease risk. In the present study dietary phytosterols and phytostanols (2.0 g/kg diet) were found, in the absence of salt-loading, to significantly increase systolic and diastolic blood pressure (*P *< 0.05, Table 4), to increase phytosterol and phytostanol accumulation in plasma, red blood cells, liver, aorta and kidney (*P *< 0.05), and to decrease cholesterol levels in liver, aorta, and kidney as well as plasma (only by phytostanols) (*P *< 0.05) (Table 3). These results suggest that, even though phytosterols and phytostanols lower cholesterol levels, their excessive accumulation in plasma and tissues may increase blood pressure thereby causing their net beneficial effects on cardiovascular events to remain questionable.

Our previous study indicated that a deficiency of membrane cholesterol due to phytosterol intake increases cell membrane rigidity, which might be a factor contributing to the shortened life span of SHRSP rats [29]. Hamano *et al*. [45] also reported that a diet containing cholesterol (10 g/kg diet) delayed the onset of stroke and prolonged the life span of SHRSP rats, whereas diets with no added cholesterol greatly shortened post-stroke survival. In the present study supplementation of phytosterols and phytostanols produced significant decreases in the ratios of cholesterol to phytosterols and phytostanols in plasma, red blood cells, liver, aorta and kidney (Table 3) and increased blood pressure (Table 4) in WKY *inbred *rats. Therefore, the increased blood pressure caused by phytosterols and phytostanols in WKY *inbred *rats may be due to increased cell membrane rigidity. Other studies, in WKY rats in the presence of salt and in SHRSP rats in the absence of salt, have proposed that enhanced membrane activity of Na+, K+-ATPase by phytosterols in rapeseed oil may play a role in elevated blood pressure [21,22]. Other mechanisms, such as endothelin and natriuretic peptides [46], have not been excluded from playing a role in hypertension caused by phytosterols and phytostanols in WKY *inbred *rats in this study and could be investigated in future studies. The molecular mechanisms of action of dietary phytosterols and phytostanols responsible for the absorption and accumulation of cholesterol, phytosterols and phytostanols have been previously discussed in our recently published paper [47].

Interestingly, it is generally speculated that phytostanol supplementation may be safer than phytosterol supplementation because the absorption of phytostanols (1-2%) is less than that of phytosterols (4-13%) [48] and the plasma levels of phytostanols are much less than that of phytosterols in humans after consumption of similar levels of either substance [49]. However, in our present study, the phytostanol diet resulted in a significant increase in diastolic blood pressure compared to the phytosterol diet (*P *< 0.05, Table 4). This result is consistent with our previous study in which SHRSP rats fed phytostanol supplemented margarine had a slightly lower survival rate than rats fed phytosterol supplemented margarine [28].

## Conclusion

Our study demonstrated for the first time that, in the absence of salt-loading, dietary phytosterols and phytostanols (2.0 g/kg diet) significantly increase systolic and diastolic blood pressure, and decrease the ratios of cholesterol to phytosterols and phytostanols in plasma, red blood cells, liver, aorta and kidney in WKY *inbred *rats. The renin-angiotensin-aldosterone system, prostanoids system, nitric oxide system, Spondin 1 and THUMP domain containing 1 do not appear to contribute significantly to the increased blood pressure. Increased cell membrane rigidity caused by a decreased ratio of cholesterol to phytosterols and phytostanols may contribute to the increased blood pressure. The net beneficial effect of phytosterols and phytostanols on cardiovascular events for individuals predisposed to over absorption of phytosterols and phytostanols requires further investigation.

## List of abbreviations

*Ace1, Ace2*: angiotensin I converting enzyme 1, 2; *Agtr1a*: angiotensin II receptor type 1a; *Nos1, Nos3*: nitric oxide synthase 1, 3; SHR: spontaneously hypertensive rats; SHRSP: stroke-prone spontaneously hypertensive rats; WKY *inbred*: Wistar Kyoto *inbred*.

## Competing interests

The authors declare that they have no competing interests.

## Authors' contributions

QC contributed to the animal feeding phase, blood pressure and gene expression measurements, data analysis as well as the drafting of the paper; HG contributed to the animal feeding phase and blood pressure measurements; ES and KC contributed to gene expression measurements; CP contributed to sterol measurements; WMNR and KAS contributed to the design of the study, data interpretation and drafting the paper. All authors read and approved the final manuscript.
